# Possible Mpox Protection from Smallpox Vaccine–Generated Antibodies among Older Adults 

**DOI:** 10.3201/eid2903.221231

**Published:** 2023-03

**Authors:** Iván Sanz-Muñoz, Laura Sánchez-dePrada, Javier Sánchez-Martínez, Silvia Rojo-Rello, Marta Domínguez-Gil, Cristina Hernán-García, Virginia Fernández-Espinilla, Raúl Ortiz de Lejarazu-Leonardo, Javier Castrodeza-Sanz, José María Eiros

**Affiliations:** National Influenza Centre, Valladolid, Spain (I. Sanz-Muñoz, L. Sánchez-dePrada, J. Sánchez-Martínez, S. Rojo-Rello, M. Domínguez-Gil, C. Hernán-García, V. Fernández-Espinilla, R. Ortiz de Lejarazu-Leonardo, J. Castrodeza-Sanz, J.M. Eiros);; Instituto de Estudios de Ciencias de la Salud de Castilla y León, Soria, Spain (I. Sanz-Muñoz, J. Sánchez-Martínez);; Hospital Clínico Universitario de Valladolid Microbiology Unit, Valladolid (S. Rojo-Rello, J.M. Eiros);; Hospital Universitario Río Hortega Microbiology Unit, Valladolid (M. Domínguez-Gil, J.M. Eiros);; Hospital Clínico Universitario de Valladolid Preventive Medicine and Public Health Unit, Valladolid (C. Hernán-García, V. Fernández-Espinilla, J. Castrodeza-Sanz)

**Keywords:** mpox, monkeypox, smallpox, vaccinia, vaccine, immunosenescence, antibodies, sexually transmitted infections, viruses, zoonoses, Spain

## Abstract

Smallpox vaccination may confer cross-protection to mpox. We evaluated vaccinia virus antibodies in 162 persons ≥50 years of age in Spain; 68.5% had detectable antibodies. Highest coverage (78%) was among persons 71–80 years of age. Low antibody levels in 31.5% of this population indicates that addressing their vaccination should be a priority.

As the 2022 mpox outbreak spread worldwide, protection against smallpox has become a focus of interest because smallpox vaccination might provide some protection against monkeypox virus (*1*). Massive vaccination with live vaccinia virus vaccines was conducted in most countries before smallpox was eradicated in 1980 (*2*), meaning a substantial proportion of persons ≥50 years of age as of 2022 might be protected against both diseases. One suggested approach to mpox protection during the current outbreak has been to administer smallpox vaccine to close contacts of infected persons (*3*,*4*). However, before taking this approach if the outbreak spreads to additional persons, concerns need to be addressed about whether smallpox vaccination provides real cross-protection and, if so, whether protection has waned over time. 

We conducted a serologic study among 162 persons ≥50 years of age in Spain who had probably received smallpox vaccination to determine the seroprevalence of vaccinia virus antibodies (VVAbs). We included 10 unvaccinated persons <40 years of age as controls, avoiding persons 40–49 years of age to eliminate possible interference in findings from persons of those ages possibly having been immunized against smallpox in the final years of vaccination. Our aim was to ascertain the presence of residual vaccinia virus immunity among adult/elderly persons. The study was approved by the ethics committee of the Eastern Health Area of ​​Valladolid (cod: PI 22–2798) and research performed according to the Declaration of Helsinki. We obtained written informed consent from participants before sampling. 

We used the Anti-Vaccinia virus IMV/Envelop protein/H3L/p35 IgG ELISA (Alpha Diagnostic International, https://www.4adi.com) to detect IgG against the vaccinia envelope protein H3L/p35, following manufacturer specifications (Appendix). VVAb levels were expressed in units per milliliter. We stratified results by age group: 50–60, 61–70, 71–80, and >80 years. 

Seroprevalence differed by age group. We found no VVAbs among the control group. Seroprevalence increased with age, until it dropped dramatically among participants >80 years of age (Figure). The 71–80 year age group exhibited the highest seroprevalence (78.0%), the >80 year group the lowest (57.5%) (p <0.05 by χ^2^ test). We found no significant differences in median VVAbs levels between the other age groups. 

**Figure:**
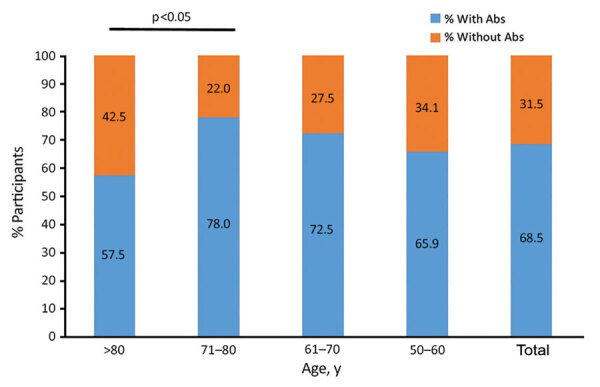
Seroprevalence of smallpox vaccine–generated antibodies among older adults, Spain. Detectable vaccinia virus antibody levels in the different age groups analyzed and for the total study population are given.

The relevance of these findings is that, 42 years after the end of routine smallpox vaccination in Spain, 68.5% of persons ≥50 years of age that we tested had detectable antibodies to vaccinia. That the highest seroprevalence was among participants in the 71–80-year age group and gradually decreased among younger age groups is probably explained by declines in smallpox vaccination coverage in Europe over time, rather than by decreased immune response. 

Although guidelines for recommended smallpox vaccination did not change during 1937–1980 in most countries in Europe, vaccination coverage in Spain and other countries declined continuously as disease eradication progressed (*5*,*6*). For example, a 2019 article reported that smallpox vaccination coverage in Guinea-Bissau fell dramatically during the 1970s, from 75% to 10%–25% (*7*). Another study, conducted in Denmark, reported that vaccination coverage dropped from 95% in 1965 to 5%–20% among persons born during the 1970s (*8*). In Spain, >6 million smallpox vaccinations were administered in 1961 but only 725,371 in 1970 and 105,573 in 1979 (*5*,*6*). Furthermore, endemic cases in high-income countries declined greatly during the 1950s (*9*). Taken together, those data illustrate that smallpox vaccination coverage steadily declined in most high-income Western countries as smallpox was increasingly confined to low-income countries (*7*). Although an imported outbreak in Yugoslavia in 1972 caused 175 cases and 35 deaths, the last nonimported case in Europe was declared in 1953 (*10*); after that date, persons became less likely to receive smallpox vaccination. 

The main limitation of our study is that we did not know the vaccination status of participants and thus could not determine whether lack of VVAbs was because of absence of vaccination or waning of antibodies. In addition, VVAbs levels might not correlate with immune protection against other orthopox viruses. The low number of participants might have affected statistical differences in results between groups. Finally, the absence of conserved cells precluded analysis of cellular immunity. 

Our findings suggest that a substantial percentage (31.5%) of persons in Spain born before 1972, especially persons born during the years when routine smallpox vaccination use waned, have either not been vaccinated against smallpox or have lost the VVAbs induced by the vaccine. Assuming 85% maximum cross-protection against monkeypox virus conferred by smallpox vaccination (*1*) and 68.5% of the population >50 years of age having detectable VVAbs, we estimated that only 58.2% of persons in those age groups would be protected. Through September 2022, a total of 813 (12.4%) mpox cases in Spain had been reported in persons >50 years of age (*11*). Limited vaccine coverage might be one cause of these cases, so vaccination against mpox or with new smallpox vaccines should be a priority in this population. 

Appendix Additional information about level of protection against mpox (monkeypox) from smallpox vaccine antibodies.
