# Supplementary material for: Possible Mpox Protection from Smallpox Vaccine–Generated Antibodies among Older Adults
Source: Emerg Infect Dis. 2023 Mar;29(3):656–8. doi: 10.3201/eid2903.221231 (PMC9973709; doi:10.3201/eid2903.221231)
Supplement: Appendix — Additional information about level of protection against mpox (monkeypox) from smallpox vaccine antibodies. [file 22-1231-Techapp-s1.pdf]

Article DOI: https://doi.org/10.3201/eid2903.221231

Possible Mpox Protection from Smallpox Vaccine–Generated Antibodies among Older Adults

Appendix

ELISA Method Used for Detecting and Quantifying Antibodies against Vaccinia Virus

A detection analysis for specific antibodies against Vaccinia poxvirus was performed to detect and quantify the antibodies against the Vaccinia virus present in the serum samples persons included in the study. To perform this analysis, the Human Anti-Vaccinia Virus (VACV) IMV/Envelope Protein/H3L/p35 IgG ELISA Kit reagents (Alpha Diagnostic International, https://www.4adi.com) were used, which enable the detection and quantification of IgG antibodies against the vaccine envelope protein H3L/p35 of the intracellular mature virion (IMV), which is the most abundant infectious protein of Orthopoxviruses. Additionally, to the serum samples included in the study, a positive and negative control (supplied with the reagents) and four calibrators (H3L positives at different concentrations) were included in duplicate to evaluate the level of vaccinia virus antibodies (VVAb) present in the positive samples.

Briefly, this method is an ELISA where the antibodies present in the serum bind to the H3L/p35 protein that coats the wells. After an initial dilution of the serum following the manufacturer's specifications (1:50), then were incubated for 60 minutes at room temperature and subsequently four washes performed. A second incubation was performed in which the secondary Anti-Human IgG HRP antibody was added and incubated at room temperature for 30 minutes. Then, the TMB chromogenic substrate (tetramethylbenzidine) was added and incubated for 15 minutes in the dark. Following this, the stop solution was added to wells and read on a plate reader at 450nm wavelength.

Reading the results was following the manufacturer´s protocol. Briefly, a standard curve was made using the duplicate values of the calibrators (1 units/mL [U/mL], 2.5 U/mL, 5 U/mL,

and 10 U/mL), expressing the antibody levels of each serum in U/mL. According to manufacturer's instructions, the value 1 U/mL was the positive threshold. Thus, those antibody values above 1 U/mL were considered positive and below as negative. The nonvaccinated controls were used to validate the established 1 U/mL cutoff value of the technique.

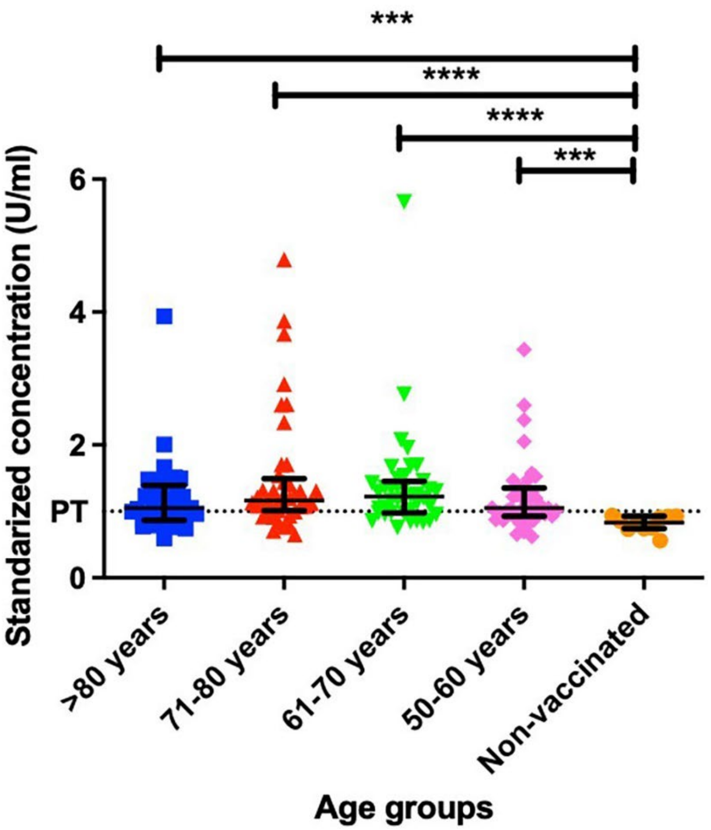

Appendix Figure. Seroprevalence of smallpox vaccine–generated antibodies among older adults, Spain. Median (interquartile range) of the standardized VVAb levels (U/mL) in each age group and in persons <40 years of age (controls) are shown. Blue boxes, >80 years of age; red upward triangles, 71–80 years of age; green downward triangles, 61–70 years of age; purple diamonds, 50–60 years of age; orange dots, nonvaccinated <40 years of age. Abs, antibodies; PT, positive threshold. *p<0.05; ***p<0.001; ****p<0.0001.
